# DEPRESSIVE SYMPTOMS AND SUBJECTIVE MEMORY AMONG MIDDLE-AGED AND OLDER MIDWESTERN COUPLES

**DOI:** 10.1093/geroni/igac059.2963

**Published:** 2022-12-20

**Authors:** Lisa Thrane, Dan Russell, Rachael Turner, Peter Rendell, Jyoti Savla, Celinda Reese-Melancon, Jennifer Margrett

**Affiliations:** Iowa State University, Ames, Iowa, United States; Iowa State University, Ames, Iowa, United States; Oklahoma State University, Stillwater, Oklahoma, United States; Australian Catholic University, Fitzroy, Victoria, Australia; Virginia Tech, Blacksburg, Virginia, United States; Oklahoma State University, Stillwater, Oklahoma, United States; Iowa State University, Ames, Iowa, United States

## Abstract

Older adults face unique challenges with the COVID-19 pandemic and its effects on mental health and cognition (Lebrasseur et al., 2021). Across the age spectrum, depression is associated with self-reported forgetfulness (Loprinzi, 2018; Ronnlund et al., 2011), and this trend is consistent among older adults with mild cognitive impairment (Ryu et al., 2016). There is some suggestion of gender differences in prospective and retrospective memory ratings among couples (Smith et al., 2000). However, little is known about how depression relates to perceptions of everyday memory performance among couples. In the current study, 47 married or cohabiting couples completed a baseline survey. The mean age of both women and men was 62 years. An Actor-Partner Interdependence Model with latent variables was employed to examine depressive symptoms with an adapted version of the Center for Epidemiological Studies-Depression scale (CES-D; Radloff, 1977) and responses to the Prospective and Retrospective Memory Questionnaire (PRMQ; Smith et al., 2000). Both constructs were parceled separately by gender and summed prior to the analysis. For women, preliminary findings revealed that higher levels of depressive symptoms were related to a negative perception of their own memory (β = .28). Conversely, men’s memory abilities were not associated with their depressive symptoms. Lastly, higher levels of depressive symptoms in men were associated with an increase in women’s self-reported forgetfulness (β = .24). These findings are suggestive of a “couple-oriented” effect (Kenny et. al., 2006), whereas there was not a statistically significant relationship between women’s depressive symptomology and men’s cognitive functioning.

